# Physical Activity Moderates Associations Between Sleep and Cognition in Middle-aged and Older Adults

**DOI:** 10.1093/geroni/igaf122.523

**Published:** 2025-12-31

**Authors:** Marc Kaizi-Lutu, Daniel Callow, Sanjana Subramanyan, Casandra Nyhuis, William Eaton, Brion Maher, Cynthia Munro, Adam Spira

**Affiliations:** Johns Hopkins Bloomberg School of Public Health, Baltimore, Maryland, United States; Johns Hopkins School of Medicine, Baltimore, Maryland, United States; Johns Hopkins Bloomberg School of Public Health, Baltimore, Maryland, United States; Penn State College of Medicine, Hershey, Pennsylvania, United States; Johns Hopkins Bloomberg School of Public Health, Baltimore, Maryland, United States; Johns Hopkins Bloomberg School of Public Health, Baltimore, Maryland, United States; Johns Hopkins School of Medicine, Baltimore, Maryland, United States; Johns Hopkins Bloomberg School of Public Health, Baltimore, Maryland, United States

## Abstract

Higher sleep quality and greater physical activity are linked to reduced risk of cognitive decline and dementia, but little is known about the synergistic effects of sleep and physical activity on cognition in middle-aged and older adults. We examined whether physical activity moderated the association between sleep and cognition in 341 cognitively unimpaired middle-aged and older adults (mean 67.5±7.40 years, 63.6% female, 35.5% non-white, 12.9±2.3 years of education), who participated in Wave 5 of the Baltimore Epidemiologic Catchment Area Study. Sleep and physical activity measures derived from 6.8±1.6 days of wrist actigraphy included total volume of physical activity (TVPA), total sleep time (TST), sleep onset latency (SOL), sleep efficiency (SE; % time in bed spent asleep) and wake after sleep onset (WASO; minutes awake after falling asleep). Cognitive tests assessed memory, executive function, language, attention, and psychomotor speed. After adjustment for age, sex, race, and education, TVPA was positively associated with attention and language. Lower SE, greater WASO, longer SOL, and TST < 6 hours (vs. TST 6-8 hours) were associated with poorer cognitive performance. TVPA moderated the associations between SOL and executive function (interaction p < 0.05), and WASO and language (interaction p = 0.04), such that associations between lower sleep quality and poorer cognition were weaker among participants with higher TVPA. Higher TVPA was associated with better language performance among those with 6-8 or > 8 hours of TST, but not in those with TST < 6 hours. Findings suggest that greater physical activity may attenuate the negative impact of poor sleep on cognition.

